# Caregivers influence preferred place of death for patients with an advanced cancer

**DOI:** 10.1017/S1478951524001858

**Published:** 2025-02-25

**Authors:** Chetna Malhotra, Shravya Murali, Isha Chaudhry

**Affiliations:** 1 Health Services and Systems Research, Duke-NUS Medical School, Singapore, Singapore; 2 Lien Centre for Palliative Care, Duke-NUS Medical School, Singapore, Singapore

**Keywords:** Advanced cancer, cohort studies, family caregivers, patient preference

## Abstract

**Objectives:**

Family caregivers influence realization of home death among advanced cancer patients. However, little is known about the caregiver factors influencing patients’ preferred and actual place of death. We aimed to assess caregiver factors associated with both caregivers’ and patients’ preferred place of death, and the association between their preferred and actual place of death.

**Methods:**

From a prospective cohort of 600 patients with stage IV solid malignancy, and 311 caregivers, we analyzed data for 227 patient–caregiver dyads of deceased patients who responded to the question on preferred place of death for patients at least once within the last 3 years before death. We assessed the association of patients’ and caregivers’ preferred place of death for patients with caregivers’ competency, employment, relationship quality with the patient, their relationship with the patient, family support, and the presence of a domestic helper. We controlled for relevant patient factors and utilized the actor–partner interdependence framework for analysis.

**Results:**

Overall, 67% patients and 74% caregivers preferred a home death for patients during the last 3 years prior to patient’s death. Patients whose caregivers reported greater caregiving competency were more likely to prefer a home death (average marginal effect: 0.02; 95% confidence interval, 0.003–0.04). Spousal caregivers were less likely to prefer a home death (−0.10 (−0.19, −0.004)). Caregivers lacking family support were more likely to prefer an institutional death (0.04 (0.002–0.08)). While caregivers’ preferences had a marginally significant association with patients’ actual place of death (*p*-value < 0.10), we did not find any association between patients’ preferred and actual place of death.

**Significance of results:**

Caregivers play a crucial role in shaping patients’ preferred and actual place of death. Supporting caregivers, particularly spousal caregivers, and enhancing their caregiving competency could potentially help achieve a home death for the patient.

## Introduction

Dying at home is widely regarded as a key indicator of quality end-of-life (EOL) care (Kinoshita et al. 2015). Yet, there has been a decline in the proportion of home deaths in various countries including Singapore (Tan et al. 2019), which is the focus for this study. Singapore’s 2023 Action Plan on Successful Aging aims to reduce hospital deaths from 61% to 51% over the next 5 years (Ministry of Health 2023). Given the influential role of family caregivers (henceforth referred to as “caregivers”) in making EOL decisions for patients including where patients die (Costa et al. 2016; Laidsaar-Powell et al. 2017; Shin et al. 2017), understanding caregiver-related factors that impact patients’ and caregivers’ preferences for a home death is essential for informing formulation of health-care policies and interventions to support caregivers in caring for patients at home and reducing hospital deaths.

Existing literature has extensively documented patient-related factors influencing preference for place of death (Fereidouni et al. 2021), including type of cancer (Blanchard et al. 2019; Chen et al. 2014; Howell et al. 2017), age (Blanchard et al. 2019; Blaney et al. 2011), financial difficulties (Marieberta et al. 2022), and quality of life (Gu et al. 2015). The literature also provides some indication that caregiver-factors such as caregiving competency, employment status, caregiver–patient relationship, and caregiver support may influence patients’ and caregivers’ preference for patients’ place of death. For instance, caregivers who are competent in caregiving tasks experience lower anxiety (Fereidouni et al. 2021; Teo et al. 2020) and may thus prefer a home death for their patient. On the other hand, employed caregivers experience high mental and financial stress due to loss of time and income during caregiving (Xiang et al. 2022), and thus may be less likely to prefer a home death. Conflicts between patients and caregivers lead to emotional burden and admission to formal care settings (Caroline et al., 2016), implying that the quality of patient–caregiver relationship may influence caregivers’ willingness to care for patient at home (Kinoshita et al. 2015). Moreover, studies suggest that spousal patients spend more days at home during EOL (Ailshire et al. 2021; Bjørnelv et al. 2020). Lastly, the presence of caregiver support may influence preferences for place of death and actual place of death by bolstering caregivers’ well-being (Choi et al. 2005; Lee and Lee 2022). To further support caregiving, an increasing number of families in Singapore employ a domestic helper, typically a woman from a low-income neighboring country to assist with various household needs, including caregiving (Østbye et al. 2013). Having a domestic helper at home could thus be associated with a greater preference for home death.

While informative, only a few existing cross-sectional studies have employed a dyadic approach to systematically assess the caregiver-related factors influencing preferences for patients’ place of death (Gu et al. 2015; Tang et al. 2010; Marieberta et al. 2022). Moreover, as patients’ clinical conditions evolve, patients’ and caregivers’ preferences regarding patients’ care, and the role of caregivers in the decision-making process may also evolve. Longitudinal dyadic data are thus crucial for capturing these changes.

In light of the significant role of caregivers and the gaps in the existing literature, our primary aim was to assess the caregiver factors associated with caregivers’ and patients’ preferences for place of death using longitudinal dyadic data. We use an actor–partner interdependence (API) model that accounts for the interdependence within dyads, estimating the effect of 1 dyad member’s characteristics on their own (actor effect) and the other dyad’s member’s (partner’s) outcomes (Cook and Kenny 2005). We hypothesized that caregivers with a greater caregiving competency, those employed, having a better relationship quality with the patient, spousal caregivers, those receiving family support, and those having a domestic helper will be more likely to prefer a home death for the patient. We also hypothesized that patients of these caregivers will be more likely to prefer a home death. Our secondary aim was to examine the extent to which patients’ and caregivers’ preferred place of death was associated with patients’ actual place of death, recognizing that not all cancer patients are able to die at their preferred place (Barclay and Arthur 2008; Howell et al. 2017).

## Methods

### Study setting and participants

A total of 600 advanced cancer patients and 345 primary caregivers were recruited for Cost of Medical Care of Patients with Advanced Serious Illness in Singapore (COMPASS), a prospective cohort study that started in July 2016. Patients were enrolled from outpatient clinics of oncology departments of 2 specialty cancer centers. Singapore citizens and permanent residents above the age of 21, diagnosed with advanced solid cancer (stage IV), and Eastern Cooperative Oncology Group (ECOG) performance status ≤2 were recruited. The caregivers recruited were the primary caregivers of the patients – defined as (1) one of the main persons providing care to the patient (e.g. accompanying patient for doctor’s visits, helping the patient’s daily activities), or (2) one of the main persons ensuring provision of care (e.g. supervision of those who provide care, such as foreign domestic workers, which is a common practice in Singapore for patients at the EOL), or (3) main person or one of the main persons involved in making treatment decisions on behalf of the patient (Harding 2013). Foreign domestic helpers were excluded as caregivers for the study. Details of the study protocol have been published (Teo et al. 2018).

Participating patients and caregivers provided written informed consent. The consent forms, surveys, and screeners for patients and caregivers were available in English, Mandarin, and Malay, and were administered in the participant’s preferred language. Patient and caregiver data were recorded via an online survey platform. Follow-up assessments were carried out every 3 months at locations preferred by the participants (i.e. outpatient clinic, home, or step-down care institution) to lower attrition rates. The study was approved by the SingHealth Centralised Institutional Review Board.

The current study utilizes data from 6-monthly follow-ups of patient–caregiver dyads of patients who had died between November 2016 and March 2022, and who had answered at least 1 survey during the last 3 years prior to the patient’s death.

### Study measures

#### Outcomes

Outcomes included preferred place of death of patients and caregivers, and the actual place of death of patients. While the participants were followed up every 3 months, preferences for place of death were assessed at baseline and subsequently at every 6-monthly follow-up to minimize response burden. Participants were asked where they would like the patient to be during the last days of life. We categorized the responses as home, institution (hospital, nursing home or hospice), or unclear (encompassing those responding as “doesn’t matter” or “any other place” without specifying a particular place). We determined actual place of death through medical health records, death certificates, and bereaved caregiver reports.

#### Main independent variables

As the outcome of preferences in place of death was assessed every 6 months, the current study utilizes study measures recorded at 6-monthly follow-ups. Caregivers’ surveys assessed their caregiving competency, employment, relationship quality with patient, actual relationship with patient, family support, and domestic helper.

We measured caregivers’ competency through the Caregiver Competence Scale (Pearlin et al. 1990; Skaff et al. 1996) consisting of 4 items (Teo et al. 2020) – caregiver’s belief in dealing with the difficult situations in caring for the patient, self-perception of caregiving, competency in caregiving skills, and confidence in caregiving. Response options were based on 4-point Likert-type scale varying from not at all competent (0) to very competent (3). Responses for all the items were added up to compute a score varying from 0 to 12, whereby a higher score indicated a better self-perception of competency.

To determine employment, caregivers were asked whether they were currently working, and their responses were categorized as employed (working full or part-time) and not employed (not employed/retired/homemaker). We assessed caregivers’ relationship quality with patient through a scale used in University of Southern California Longitudinal Study of Three-Generation Families (Lawrence et al. 1998). The scale comprised of 4 items, and each item was rated on a 4-point Likert-type scale. The responses for all the questions were summed to calculate a score varying from 0 to 12, with a higher score indicating a better relationship quality. Caregivers’ actual relationship with the patient was coded as spouse or non-spouse.

We assessed caregivers’ lack of family support using 5 items from the lack of family support subscale of the Caregiver Reaction Assessment (Given et al. 1992; Malhotra et al. 2012). The items were coded on a 5-point Likert ranging from 0 (strongly disagree) to 4 (strongly agree). Items were averaged to generate a total score ranging between 1 and 5 with a higher score indicating poorer family support (Kristanti et al. 2021). Lastly, we asked the caregivers if they had any additional help – helper/maid/foreign domestic worker – to take care of the patient (yes/no).

#### Covariates

We controlled for relevant patient factors including the type of cancer, time from their death, age (as obtained from medical records), quality of life (as measured by Functional Assessment of Cancer Therapy-General (FACT-G; Cella et al. 1993)), and financial difficulties. Patients’ financial difficulties were assessed through 3 questions on how well the amount of money (from all the sources including their earnings, savings, etc.) (1) enables them to cover the cost of their treatment; (2) allows them to take care of their daily needs; (3) enables them to buy small luxuries. Responses for each question were categorized as (1) very well; (2) fairly well; or (3) poorly.

All scales used in this study have been validated and used previously in Asian contexts (Aloweni et al. 2019; Chan et al. 2018; Koh et al. 2022; Østbye et al. 2013; Teo et al. 2020). They were used in the appropriate language versions (English/Malay/Mandarin) as available by the developer. If a specific language version was not available, it was translated by bilingual speakers based on the developer instructions. Furthermore, pilot interviews were conducted prior to commencing the main survey to assess the face validity of the survey responses. Lastly, we checked the internal consistency reliability for all scales used in the current analysis using Cronbach’s alpha (Supplementary Table 1).

#### Statistical analyses

The sample size calculations for the full cohort study have been previously published (Teo et al. 2018). A post-hoc power calculation showed that analytic sub-sample of 1,168 observations for 227 dyads was adequate to run a multivariable logistic regression model (sensitivity analysis for preference for home versus non-home death) with continuous independent variables. We conducted this post-hoc power analysis using “*powerlog*” package in Stata that uses the estimated probability of outcome (=1, i.e. home death) at the mean and standard deviation of the independent variable and considers the multiple correlation between all independent variables. To achieve 80% power, we needed 153 dyads, therefore, our sample size of 227 dyads was considered adequate for the analysis.

We used Stata 17 for all the analyses (StataCorp 2021). We described the preferred place of death among patient–caregiver dyads in the last 3 years of life prior to patient’s death. We performed a test of proportions to compare patients’ and caregivers’ preferences for institutional and home death at the start and end of the study period. As preferences for patient’s death was elicited at every 6-monthly follow-up, we utilized up to 7 surveys for patients and caregivers during the study period. Overall, 71 dyads had responded to only 1 survey, 60 dyads had responded to 2 surveys, 96 dyads had responded to at least 3 surveys in the last 3 years prior to patient’s death. We used all available surveys in the analysis.

We used an API framework (Cook and Kenny 2005) to jointly analyze patients’ and caregivers’ preferred place of death (dying at home, institution or unclear) whereby members of the dyad were assumed to be non-independent, and the dyad rather than individuals were treated as a unit of analysis. It has been used previously in context of analyzing dyadic data from spouses (Karademas 2014), parents and children (Pesonen et al. 2006), as well as romantic partners (Pollard et al. 2014). However, it has not been widely used in context of patient–caregiver dyadic data, despite the influence of caregivers on patient outcomes (Edwards and Ruettiger 2002; Hahn-Goldberg et al. 2018; Malhotra et al. 2021).

We implemented the API framework using a mixed-effects multinomial logistic regression. Mixed-effects regression modeling systematically accounts for fixed and random effects representing variability at both the item-level (within participants) and subject-level (across participants) in the longitudinal analysis (Aggrey 2009). It utilizes all available data from repeated measurements over time and uses maximum likelihood estimation to handle data missing at random (Pugh et al., 2022). Details about how our dataset was structured are in the Supplement.

The independent variables used in the analyses were the role of the respondent (patient/caregiver), and those described above (caregiver competency, employment, relationship quality with patient, type of relationship with the patient, lack of family support, and having a domestic helper). We controlled for relevant patient factors including time from patient’s death, cancer type, patient age, quality of life, and financial difficulties. To estimate the actor and partner effect, we assessed the interaction between respondent role with each independent variable. We estimated average marginal effect (AME) that reflected the average change in predicted values of the outcome with a unit change in each independent variable (Jann 2013). We included dyadic identification number as a random effect. The value of AME indicates the average of the change in predicted probability of patients/caregivers preferring a particular place of death (e.g. home death) with 1 unit change in the independent variable (e.g. caregiver competency score).

We conducted a sensitivity analysis with preferred place of death as a dichotomous outcome (home versus others) and the same set of independent variables described earlier using a mixed-effects logistic regression model.

Lastly, we used a logistic regression model to assess the association between patients’ actual place of death (dependent variable – home death vs others) and the patients’ and caregivers’ preferred place of death at the last assessment.

## Results

### Patient characteristics

A total of 600 patients enrolled in the study consented to participate in the survey questionnaire, of which 289 (48%) were recruited without a caregiver and were excluded from the study. Of the remaining 311 eligible patient–caregiver dyads, 227 patients who were deceased, and had answered at least 1 survey in last 3 years prior to patient’s death, constituted our analytic sample (Supplementary Figure 1). The sample patient and caregiver characteristics at the earliest assessment in the last 3 years prior to patient’s death are described in Table 1.

**Table 1.** Patient and caregiver characteristics at the baseline (*n* = 227)

Patient characteristics	
Age, mean (SD)	62.6 (10.3)
Gender, *n* (%) Female Male	111 (48.9) 116 (51.1)
Months from death, *n* (%) 6 months and below 6–12 months 12–18 months 18–24 months 24–30 months 30–36 months	139 (23.2) 159 (26.5) 119 (19.9) 73 (12.2) 65 (10.9) 44 (7.4)
Cancer type, *n* (%) Breast Colorectal/Gastrointestinal Genitourinary/Gynecologic Respiratory Others	28 (12.3) 65 (28.6) 50 (22.0) 63 (27.8) 21 (9.3)
Caregiver characteristics	
Age, mean (SD)	49.8 (9.0)
Gender, *n* (%) Female Male	143 (63.0) 84 (37.0)
Caregiver relationship with patient, *n* (%) Spouse Others	111 (48.9) 116 (51.1)
Currently employed, *n* (%) Yes No	85 (37.4) 142 (62.6)
Quality of relationship with patient (range, 0–12), mean (SD)	9.0 (2.4)
Competency (range, 2–12), mean (SD)	8.8 (2.0)
Lack of family support (range, 1–5), mean (SD)	2.3 (0.6)
Presence of foreign domestic helper, *n* (%) Yes No	58 (25.6) 169 (74.5)

### Preferred place of death

**Figure 1.**
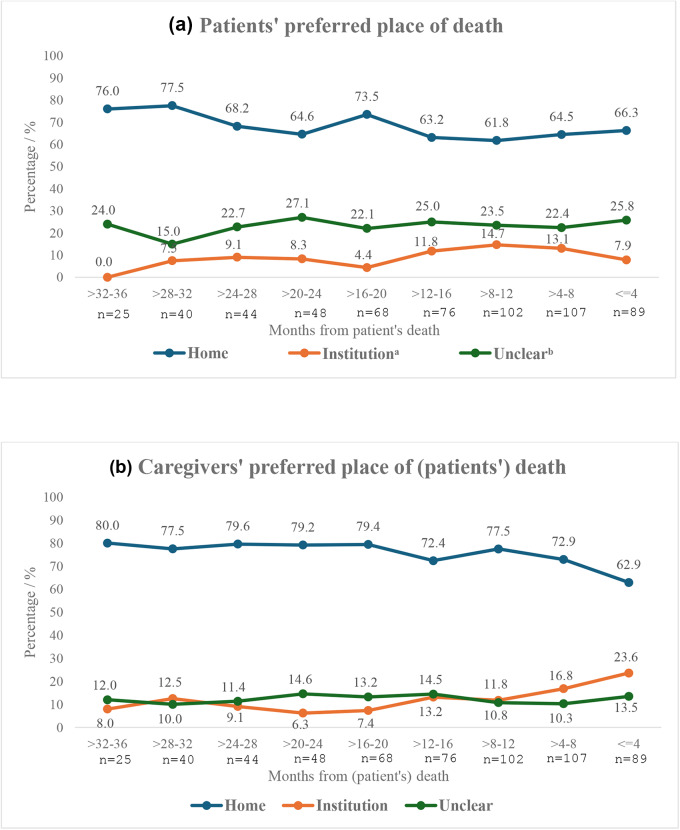
Patients’ and caregivers’ preferred place for (patients’) death during the last 3 years of patients’ life, *n* = 227.

^a^Includes both hospitals and hospice.^b^Includes others or an unclear preference.

Overall, most patients (66.8%) and caregivers (74.5%) preferred home death for patients in the last 3 years prior to patient’s death. At the earliest assessment (i.e. 32–36 months prior to patient’s death), 80% of caregivers preferred a home death, and in the last 4 months prior to death, 62.9% preferred a home death (*p*-value > 0.1). Furthermore at the earliest assessment, 8% preferred an institutional death, and in the last 4 months prior to death, 23.6% preferred an institutional death (*p* = 0.09) (Figure 1). Among patients, nearly three-quarter (76%) preferred a home death at the earliest assessment, and 66% preferred it in the last 4 months (*p*-value > 0.10).

### Factors associated with the preferred place of death

**Figure 2.**
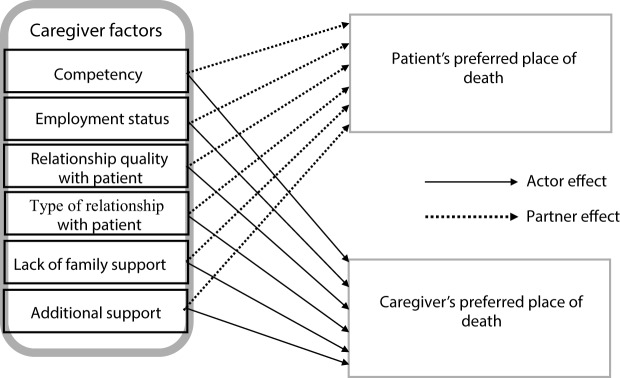
Conceptual framework showing that caregiver factors are associated with their own and the patient’s preferred place of death.

Figure 2 shows the API framework used to analyze the dyad data. Correlation between all independent variables was <0.5 (Supplementary Table 2), therefore reducing multicollinearity and the risk of faulty inferences (Farrar and Glauber 1967). Table 2 shows that patients whose caregivers reported greater caregiving competency were more likely to prefer a home death (AME, 0.02; 95% CI, 0.003–0.04), while those whose caregivers reported lower caregiving competency were more likely to have an unclear preference (−0.02 (−0.03 to 0.001)). Caregivers with lack of family support were more likely to prefer an institutional death (0.04 (0.002–0.08)). Contrary to our hypothesis, spousal caregivers were less likely to prefer a home death (−0.10 (−0.19, 0.004)), and more likely to have an unclear preference for patient’s place of death (0.11 (−0.04 to 0.18)) (Table 2).

**Table 2.** Association of caregiver factors with dyads’ preferred place of death, *n* = 227

		Patient		Caregiver	
Caregiver factor	Preferred place of death	Average marginal effect (95% confidence interval)	*p*-value	Average marginal effect (95% confidence interval)	*p*-value
Competency	Home	**0.02 (0.003, 0.04)**	**0.03**	0.006 (−0.01, 0.02)	0.49
	Institution	−0.006 (−0.02, 0.006)	0.37	−0.006 (−0.02, 0.008)	0.42
	Unclear	−0.02 (−0.03, 0.001)	0.07	−0.001 (−0.02, 0.01)	0.93
Employment status	Home	0.03 (−0.07, 0.12)	0.56	−0.01 (−0.10, 0.07)	0.74
	Institution	0.004 (−0.05, 0.06)	0.88	0.005 (−0.06, 0.07)	0.89
	Unclear	−0.03 (−0.12, 0.05)	0.46	0.01 (−0.06, 0.08)	0.77
Relationship quality with patient	Home	−0.006 (−0.02, 0.01)	0.50	0.01 (−0.004, 0.03)	0.14
	Institution	0.003 (−0.008, 0.01)	0.62	−0.004 (−0.02, 0.008)	0.48
	Unclear	0.003 (−0.013, 0.02)	0.68	−0.008 (−0.02, 0.004)	0.20
Type of relationship with patient	Home	0.04 (−0.06, 0.14)	0.43	**−0.10 (−0.19, −0.004)**	**0.04**
	Institution	0.007 (−0.05, 0.06)	0.81	−0.016 (−0.08, 0.05)	0.61
	Unclear	−0.05 (−0.14, 0.04)	0.32	**0.11 (−0.04, 0.18)**	**0.003**
Lack of family support	Home	−0.03 (−0.08, 0.04)	0.41	−0.03 (−0.08, 0.03)	0.34
	Institution	0.02 (−0.02, 0.06)	0.33	**0.04 (0.002, 0.08)**	**0.04**
	Unclear	0.008 (−0.05, 0.06)	0.79	−0.02 (−0.06, 0.03)	0.50
Additional support	Home	0.011 (−0.08, 0.10)	0.82	−0.02 (−0.11, 0.06)	0.56
	Institution	−0.05 (−0.11, 0.01)	0.11	−0.01 (−0.08, 0.05)	0.72
	Unclear	0.04 (−0.04, 0.12)	0.36	0.04 (−0.03, 0.10)	0.25

Values highlighted in bold indicates *p*-value < 0.05.

### Sensitivity analyses

Sensitivity analysis results showed significant association between caregiver competency and patients’ preference for home death, as well as between the caregiver’s relationship with the patient and caregivers’ preference of home death for patients (Table 3).

**Table 3.** Mixed effect logistic regression estimates: Association of caregiver factors with dyads’ preferred place of death, *n* = 227

Caregiver factor	Patient		Caregiver	
	Average marginal effect^a^ (95% confidence interval)	*p*-value	Average marginal effect (95% confidence interval)	*p*-value
Competency	**0.02 (0.003, 0.04)**	**0.02**	0.007 (−0.01, 0.03)	0.47
Employment	0.03 (−0.07, 0.12)	0.55	−0.01 (−0.10, 0.07)	0.78
Relationship quality with patient	−0.007 (−0.025, 0.012)	0.48	0.01 (−0.004, 0.03)	0.15
Lack of family support	−0.02 (−0.08, 0.04)	0.50	−0.02 (−0.08, 0.03)	0.40
Additional support	0.008 (−0.09, 0.10)	0.87	−0.03 (−0.12, 0.05)	0.44
Type of relationship with patient	0.04 (−0.06, 0.14)	0.39	**−0.009 (−0.18, −0.004)**	**0.041**

a Average marginal effects (AMEs) from mixed effects logistic regression estimates.Values highlighted in bold indicates *p*-value < 0.05.

### Association with preferred and actual place of death

We did not find any significant association between patients’ preferred places of death at the last assessment, and their actual places of death. The association between caregiver’s preferred place of death at the last assessment and their patient’s actual place of death approached statistical significance (*p* = 0.09) but was not statistically significant at a 5% level (Table 4). Of the caregivers who preferred an institutional death (*n* = 38) at the last assessment, 63% of their patients died at an institution. Among caregivers who preferred home death (*n* = 158), only 35% of their patients died at home. Of the patients who preferred an institutional death at the last survey (*n* = 25), 76% died at an institution while among patients who preferred a home death (*n* = 146), only 34% of them died at home.

**Table 4.** Association between patient and caregiver’s preferences in home death and patients’ actual home death

Predictors	Coefficient (95% confidence interval)	*p*-value
Patient’s preference for home death	0.03 (−0.57, 0.63)	0.91
Caregiver’s preference for home death	0.56 (−0.09, 1.22)	0.09

## Discussion

Using the 6-monthly follow-up data of a prospective cohort of 227 patient–caregiver dyads who were surveyed until patient’s death, we showed that more than two-thirds of the patients and caregivers preferred home death in the last 3 years prior to patient’s death, and nearly a quarter of caregivers (24%) preferred an institutional death in the last 4 months prior to their patient’s death. We also found that while patients’ preferences were not associated with the patients’ actual place of death, caregivers’ preferences may be associated with the patients’ actual place of death with results approaching statistical significance at 10% level. Caregivers who lacked family support were more likely to prefer an institutional death (versus dying at home or having an unclear preference), and patients whose caregivers reported greater caregiving competency were more likely to prefer a home death (versus dying in an institution or having an unclear preference). Furthermore, a majority of patients and caregivers had their preferences for an institutional death met (as reported in their last survey prior to death), while only about a third of patients and caregivers had their preferences for a home death met. Our findings have implications for supporting caregivers in caring for patients at home.

Our study found that while most patients preferred a home death through the period of study, we observed an increasing proportion of caregivers preferring an institutional death in the last 4 months prior to their patient’s death. Patients’ preference for a home death is likely to be related to the positive feelings they associate with their home such as familiarity, safety, and historical meaning (Costa et al. 2016; Milligan et al. 2016). However, caregivers may consider the worsening symptom burden of patients toward death, and may believe that institutions offer more effective management of symptoms and constant professional care – services that may be unavailable at home (Brazil et al. 2005).

We noted greater caregiving competency to be associated with patients’ preference for a home death, and a lower caregiving competency to be associated with patients’ unclear preference. Lower caregiver competency has previously been found to be associated with greater anxiety among caregivers (Teo et al. 2020). Patients may sense their caregivers to be anxious, may perceive themselves to be a burden to their caregivers, and thus may be less likely to report a preference for home death. Contrary to our hypothesis, we did not find caregivers’ competency to be associated with their own preference. The reasons for this need to be further investigated.

Moreover, a lack of family support for caregivers was associated with a preference for an institutional death (for patients) among caregivers, but not for patients. A previous study in Singapore reported that dementia caregivers who lacked family support expressed greater frustration and unhappiness (Basnyat and Chang 2021). Caregivers for cancer patients in this study may be experiencing similar emotions and a high burden, and thus may seek professional support within institutions during patients’ last days of life. Caregivers may not always communicate the lack of family support and their anxiety with the person they are caring for, and thus lack of family support may not influence patients’ preferred place of death.

Compared to other family members, spousal caregivers are known to experience greater burden and anxiety about the loss of patients (Pinquart and Sörensen 2003; Savundranayagam and Orange 2011), and hence patients’ home death may impact their meaning of home (Milligan et al. 2016) as they continue to live in it. Many spousal caregivers’ preference for an institutional death for the patient may also be in conflict with an “ideal” for a home death (Lang 2020), leading to a dilemma regarding patients’ place of death and resulting in them having an unclear preference. Alternatively, many spousal caregivers may be unwilling to confront the possibility of patient’s death, resulting in an unclear preference.

Our study revealed that while there was no significant association between patients’ last preferred place of death and their actual place of death, the preference expressed by caregivers was associated with the patients’ actual place of death, approaching statistical significance at a 10% level. This suggests that caregivers may wield a more substantial influence on determining where patients spend their final moments, with similar findings reported in previous studies from Singapore (Lee et al. 2017) and other countries (McWhinney et al. 1995; Tang et al. 2005; Visser et al. 2004). Considering the small sample size of our analyses, a future study conducted with a larger sample size is needed to confirm this association.

Moreover, our results showed that a high proportion of patients and caregivers that preferred an institutional death in their last survey assessment were able to die in the institution, while preferences for a home death could not be met. This highlights the challenges of providing home care at the EOL. These findings emphasize the need for greater patient–caregiver communication regarding patient preferences for care, and supporting caregivers to help patients achieve their preferred place of death.

Our study has several implications. As highlighted in Singapore’s 2023 Action Plan on Successful Aging (Ministry of Health), there is an increasing attempt by policy makers to reduce hospital deaths and increase the proportion of home deaths over the next 5 years. Our study provides an understanding of how caregivers can be better supported in efforts to accomplish this goal. Results provide some indication that supporting caregivers, for instance through friends/family, as well as improving caregivers’ competency in caring for patients may influence preference for a home death. Future studies can develop and evaluate potential interventions to support caregivers and to improve their competency, especially for spousal caregivers.

The study has several strengths. We used prospective data from a large number of patient–caregiver dyads over time to visualize variation in preferences over the final years of life. The association between caregiver factors and preferred place of death has not been directly studied previously, especially in the Singapore context. Furthermore, our study is also novel in using an API model in assessing the association of caregiver factors with both patients’ and caregivers’ preferences. The study also has limitations. First, due to its non-experimental design, causal inference is not possible. Second, as both the outcomes and the independent variables are self-reported, the association observed could have been caused by the reporting behaviors of the patients and the caregivers. Third, our sample is not representative of advanced cancer patient–caregiver dyads. Lastly, due to cultural and familial differences, the generalizability of our study should be tested in other settings.

Yet, our study serves as a model to explore other potential EOL research questions involving dyads, such as patient–caregiver or physician–patient dyads. Dyadic approaches, such as API, are crucial as they allow us to analyze how the evolving roles of each member of the dyad in the context of serious illnesses intersect and impact the care received (Malhotra et al. 2021; Renee Jacops and Fasolino 2022). Furthermore, the members of the dyads, e.g. patients and caregivers, tend to cope with serious illnesses as a unit (Traa et al. 2015), and influence the well-being of each other as a result, which further exemplifies the need to use dyadic approaches.

In conclusion, this study shows that most patients and caregivers preferred a home death for the patient. Yet, most patients died at an institution, highlighting the challenges of providing home care at the EOL. Supporting caregivers, especially spousal caregivers, and improving their caregiving competency may increase patients’ and caregivers’ preference for patients to be at home, and ultimately the proportion of patients of achieving care consistent with their preferences.

## Supporting information

Malhotra et al. supplementary material Malhotra et al. supplementary material

## Data Availability

Data are available on reasonable request from the corresponding author.

## Supplementary material

The supplementary material for this article can be found at https://doi.org/10.1017/S1478951524001858.
