# Supplementary material for: Caregivers influence preferred place of death for patients with an advanced cancer
Source: Palliat Support Care. 2025 Feb 25;23:e41. doi: 10.1017/S1478951524001858 (PMC13166561; doi:10.1017/S1478951524001858)
Supplement: Malhotra et al. supplementary material [file S1478951524001858sup001.docx]

**Supplement**

**Description of analytical dataset structure for API Framework**

We analysed the association between patient and caregiver’s preferred place of death (study outcome) with caregiver factors (caregiver competency, employment, relationship quality with patient, type of relationship with the patient, lack of family support and having a domestic help) after controlling for patient factors (cancer type, patient age, quality of life and financial difficulties) and time from patient’s death.

We created a longitudinal dataset for our analysis. Each row of this dataset represented individual respondents, either patients or caregivers. We assigned a unique dyad identification number across the two rows, ensuring a distinct identifier for each dyad. We created a variable specifying the role of the respondent - patient or caregiver. The value of the outcome variable in the ‘patient’ row corresponded to patient preference for place of death and in the ‘caregiver’ row corresponded to caregiver’s preference.

We calculated time from death as time between date of survey assessment and patient’s death. At each timepoint, the value of ‘time from death’ was the same in both rows for the respective dyad.

The caregiver factors described above were presented as separate columns and their values for “caregiver” were used to assess the actor effect, i.e. the association of caregiver factors with caregiver’s preference for patient’s place of death. These values were then repeated for the row indicating “patient” participant role and were used to assess the partner effect of caregiver factors on patient’s preference for place of death. Thus, a longitudinal dataset was created for that included all the unique dyads, participant role, time of assessment, participant role, patient and caregiver factors used in the analysis.

**Supplementary Figure 1 – Flowchart of study participants**

Excluded (withdrew consent), n=2

Approached, n=1,137

Excluded (did not meet inclusion criteria), n=95

Eligible, n=1,042

Excluded (declined to participate), n=393

Enrolled, n=649

Patients included in the study, n=647

- Records review only (n= 47)
- Survey and records review (n= 600)

Deceased during the study period, n=229

Patient enrolled with a caregiver i.e. dyads, n=311

Excluded (only patient enrolled without a caregiver), n=338

Excluded (Patient still alive), n=82

Excluded (did not answer the survey in last 3 years of life), n=2

Dyads analyzed, n=227

**Supplementary Table 1. Reliability and internal validity of scales used in the study**

| **Scale** | **Cronbach alpha** |
| --- | --- |
| Caregiver competence scale | 0.82 |
| University of Southern California Longitudinal Study of three-generation families to measure quality of patient-caregiver relationship | 0.87 |
| Caregiver reaction assessment | 0.86 |

**Supplementary Table 2. Correlation between independent variables**

| **Variable** | **Caregiver’s**  **competency** | **Caregiver’s**  **employment status** | **Caregivers’**  **relationship quality with patient** | **Caregiver’s actual relationship with patient** | **Lack of family support for caregivers** | **Additional support for caregivers** | **Patient’s age** | **Patient’s financial difficulties** | **Patient’s cancer type** | **Patient’s quality of life** |
| --- | --- | --- | --- | --- | --- | --- | --- | --- | --- | --- |
| **Caregiver’s**  **competency** | 1.00 | - | - | - | - | - | - | - | - | - |
| **Caregiver’s**  **employment status** | -0.16 | 1.00 | - | - | - | - | - | - | - | - |
| **Caregivers’**  **relationship quality with patient** | 0.38 | -0.07 | 1.00 |  | - | - | - | - | - | - |
| **Caregiver’s actual relationship with patient** | 0.13 | -0.35 | 0.12 | 1.00 | - | - | - | - | - | - |
| **Lack of family support for caregivers** | -0.05 | 0.10 | -0.16 | -0.10 | 1.00 | - | - | - | - | - |
| **Additional support for caregivers** | 0.13 | 0.10 | -0.04 | 0.03 | 0.05 | 1.00 | - | - | - | - |
| **Patient’s age** | -0.02 | -0.17 | -0.16 | -0.17 | 0.05 | 0.03 | 1.00 | - | - | - |
| **Patient’s financial difficulties** | -0.10 | 0.07 | -0.03 | -0.13 | 0.12 | -0.02 | 0.02 | 1.00 | - | - |
| **Patient’s cancer type** | -0.09 | 0.01 | -0.04 | 0.20 | -0.10 | -0.01 | 0.00 | -0.02 | 1.00 | - |
| **Patient’s quality of life** | 0.10 | -0.05 | 0.19 | -0.03 | 0.08 | -0.10 | 0.07 | -0.31 | -0.08 | 1.00 |
